# Characteristics of intensive care unit registries - findings from the Global Registry ICU Datasets (GRID) survey

**DOI:** 10.62675/2965-2774.20260168

**Published:** 2026-01-09

**Authors:** Luigi Pisani, Paola Di Lecce, Cornelius Sendagire, Vrindha Pari, Carlo Olivieri, Rabiul Alam Md Erfam Uddin, Diptesh Aryal, Priyantha Athapattu, Sean Bagshaw, Gaston Burghi, Eirik Alnes Buanes, Steffen Christensen, Rory Dwyer, Ariel Leonardo Fernández, Stefano Finazzi, Bertrand Guidet, David Harrison, Eva Hanciles, Madiha Hashmi, Satoru Hashimoto, Nao Ichihara, Nazir I. Lone, Maria del Pilar Arias López, Yen L. Minh, Andreas Perren, Koukeo Phommasone, David Pilcher, Matti Reinikainen, Wangari Waweru-Siika, Moses Siaw-Frimpong, Martin I. Sigurdsson, Maryam Shamal, Menbeu Sultan, Jose Emmanuel M. Palo, David Thomson, Bharath Kumar Tirupakuzhi Vijayaraghavan, Abigail Beane, Rashan Haniffa, Dave A. Dongelmans, Miklos Lipcsey, Jorge Ibrain Figueira Salluh

**Affiliations:** 1 University of Bari "Aldo Moro" Department of Precision-Regenerative Medicine and Jonic Area Section of Anesthesiology and Intensive Care Medicine Bari Italy Section of Anesthesiology and Intensive Care Medicine, Department of Precision-Regenerative Medicine and Jonic Area, University of Bari "Aldo Moro" - Bari, Italy.; 2 Mahidol Oxford Tropical Research Unit Bangkok Thailand Mahidol Oxford Tropical Research Unit - Bangkok, Thailand.; 3 University of Makerere Uganda Heart Institute Makerere Uganda Uganda Heart Institute, University of Makerere, Makerere, Uganda; 4 University of Birmingham Birmingham England University of Birmingham - Birmingham, England.; 5 Sant’Andrea Hospital Anesthesia and Intensive Care Vercelli Italy Anesthesia and Intensive Care, Sant’Andrea Hospital - Vercelli, Italy,; 6 Chittagong Medical College Hospital Department of Medicine Chattogram Bangladesh Department of Medicine, Chittagong Medical College Hospital - Chattogram, Bangladesh,; 7 Department of Critical Care, Nepal Intensive Care Research Foundation Kathmandu Nepal Department of Critical Care, Nepal Intensive Care Research Foundation - Kathmandu, Nepal.; 8 Mahidol Oxford Tropical Medicine Research Unit Nat-Intensive Care Surveillance Colombo Sri Lanka Nat-Intensive Care Surveillance, Mahidol Oxford Tropical Medicine Research Unit - Colombo, Sri Lanka.; 9 University of Alberta Department of Critical Care Medicine Alberta Canada Department of Critical Care Medicine, University of Alberta - Edmonton, Alberta, Canada.; 10 Hospital Maciel Intensive Care Unit Montevideo Uruguay Intensive Care Unit, Hospital Maciel, ASSE - Montevideo, Uruguay.; 11 Norwegian Intensive Care and Pandemic Registry Bergen Norway Norwegian Intensive Care and Pandemic Registry - Bergen, Norway.; 12 Haukeland University Hospital Bergen Norway Haukeland University Hospital - Bergen, Norway.; 13 Aarhus University Hospital Department of Anaesthesia and Intensive Care Medicine Aarhus Denmark Department of Anaesthesia and Intensive Care Medicine, Aarhus University Hospital - Aarhus, Denmark.; 14 Royal College of Surgeons in Ireland National Office for Clinical Audit Dublin Ireland National Office for Clinical Audit, Royal College of Surgeons in Ireland - Dublin, Ireland.; 15 Sociedad Argentina de Terapia Intensiva Buenos Aires Argentina Quality Program, Sociedad Argentina de Terapia Intensiva - Buenos Aires, Argentina.; 16 Mario Negri Institute for Pharmacological Research Department of Medical Epidemiology Laboratory of Clinical Data Science Lombardia Italy Laboratory of Clinical Data Science, Department of Medical Epidemiology, Mario Negri Institute for Pharmacological Research - Lombardia, Italy.; 17 Collège des Utilisateurs de Bases de Données en Réanimation Paris France Collège des Utilisateurs de Bases de Données en Réanimation - Paris, France.; 18 Intensive care National Audit & Research Centre Holborn United Kingdom Intensive care National Audit & Research Centre - Holborn, United Kingdom.; 19 Connaught Hospital University of Sierra Leone Hospital Complex Freetown Sierra Leone University of Sierra Leone Hospital Complex, Connaught Hospital - Freetown, Sierra Leone.; 20 Ziauddin University Department of Critical Care Medicine Karachi Pakistan Department of Critical Care Medicine, Ziauddin University - Karachi, Pakistan.; 21 Japanese Intensive Care Patient Database Tokyo Japan Japanese Intensive Care Patient Database - Tokyo, Japan.; 22 University of Edinburgh Centre for Inflammation Research Edingurgh United Kingdom Centre for Inflammation Research, University of Edinburgh - Edingurgh, United Kingdom.; 23 Public Health Scotland Scottish Intensive Care Society Audit Group Edingurgh United Kingdom Scottish Intensive Care Society Audit Group, Public Health Scotland - Edingurgh, United Kingdom.; 24 Oxford University Clinical Research Unit Ho Chi Minh City Vietnam Clinical Research Unit, Oxford University - Ho Chi Minh City, Vietnam.; 25 Société Suisse de Médecine Intensive Schweizerische Gesellschaft für Intensivmedizin Basel Switzerland Schweizerische Gesellschaft für Intensivmedizin, Société Suisse de Médecine Intensive - Basel, Switzerland.; 26 Mahosot Hospital Intensive Care Unit Vientiane Laos Intensive Care Unit, Mahosot Hospital - Vientiane, Laos.; 27 Australian and New Zealand Intensive Care Society Clinical Quality Registry Centre for Outcome and Resource Evaluation Prahran Australia Centre for Outcome and Resource Evaluation, Clinical Quality Registry, Australian and New Zealand Intensive Care Society - Prahran, Australia.; 28 Kuopio University Hospital Department of Anaesthesiology and Intensive Care Kuopio Finland Department of Anaesthesiology and Intensive Care, Kuopio University Hospital -Kuopio, Finland.; 29 Aga Khan University Nairobi Department of Anaesthesia Nairobi Kenya Department of Anaesthesia, Aga Khan University Nairobi - Nairobi, Kenya.; 30 Komfo Anokye Teaching Hospital Department of Anaesthesiology and Intensive Care Kumasi Ghana Department of Anaesthesiology and Intensive Care, Komfo Anokye Teaching Hospital - Kumasi, Ghana.; 31 University of Iceland Faculty of Medicine Reykjavik Iceland Faculty of Medicine, University of Iceland - Reykjavik, Iceland.; 32 Wazir Akbar Khan Hospital Kabul Afghanistan General Surgery, Wazir Akbar Khan Hospital - Kabul, Afghanistan.; 33 St. Paul's Hospital Millennium Medical College Department of Emergency Medicine and Critical Care Addis Ababa Ethiopia Department of Emergency Medicine and Critical Care, St. Paul's Hospital Millennium Medical College - Addis Ababa, Ethiopia.; 34 The Medical City Acute and Critical Care Institute Pasig City Philippines Acute and Critical Care Institute, The Medical City - Pasig City, Philippines.; 35 University of Cape Town Department of Anaesthesia and Perioperative Medicine Cape Town South Africa Department of Anaesthesia and Perioperative Medicine, University of Cape Town - Cape Town, South Africa.; 36 Apollo Hospitals Educational and Research Foundation Department of Critical Care Chennai India Department of Critical Care Medicine, Apollo Hospitals Educational and Research Foundation - Chennai, India.; 37 University of Amsterdam Amsterdam Medical Center Department of Intensive Care Amsterdam The Netherlands Department of Intensive Care, Amsterdam Medical Center, University of Amsterdam - Amsterdam, The Netherlands.; 38 Uppsala University Department of Surgical Sciences Anesthesiology and Intensive Care Uppsala Sweden Anesthesiology and Intensive Care, Department of Surgical Sciences, Uppsala University - Uppsala, Sweden.; 39 Instituto D’Or de Pesquisa e Ensino Rio de Janeiro BR Brazil Instituto D’Or de Pesquisa e Ensino - Rio de Janeiro (BR), Brazil.

**Keywords:** Intensive care units, Registries, Dataset, Outcomes, Quality control, Quality improvement, Registry-enabled research, Data collection, Internet, Survey and questionnaires

## Abstract

**Background:**

Intensive care unit registries, which aim to improve the quality of intensive care unit care through benchmarking and quality improvement initiatives, are active worldwide, with considerable dishomogeneity. We aimed to map core datasets, additional variables, and research activities of these registries.

**Methods:**

A cross-sectional survey was disseminated to registry leads between October 2023 and June 2024. The survey was structured into four main topics: registry characteristics and coverage, core dataset features, additional modules, and registry-enabled research.

**Results:**

Leads of 34/42 national registries responded (response rate 81%), covering 3,337 intensive care units, with a larger representation from South America. Systematized nomenclature of medicine, clinical terms, and customized categorical classifications were the main nomenclatures used. All registries except one employed a severity of illness score/risk prediction model. The SOFA score was reported by 88% of registries. Organ support measures were often recorded, including mechanical ventilation (97%), vasopressor administration (86%) and renal replacement therapy (86%). Three out of four intensive care unit registries coded interventions such as intubations, intravenous lines and tracheostomies. Additional datasets differed, with many use cases for nosocomial infection burden, bed availability and staffing resources. Over half of intensive care unit registries had current structured quality improvement initiatives. Registry-enabled observational research was reported in 46% of registries, while interventional studies were reported in only 22%.

**Conclusion:**

Over three thousand intensive care units in 35 countries participate in an intensive care unit registry. Despite heterogeneity in coding systems, risk models, and additional datasets, we identify several areas of convergence that may inform a future shared core dataset. There is potential for further intensive care unit registry-based research, particularly interventional.

## INTRODUCTION

Intensive care unit (ICU) registries are designed to collect standardized data on patient demographics, treatments, outcomes, and resource use across different ICUs.^(1)^ Data on case-mix, resource use, and risk-adjusted outcomes allows healthcare providers to assess and benchmark performance, identify adherence to best practices, and track trends and variations in care and resource use, which helps to identify ways to improve service provision, patient outcomes and safety.^(2)^ Some registries have evolved to provide data to inform clinical research, enabling studies on the effectiveness of various interventions, the identification of risk factors, and the validation of predictive models.^(3)^ Additionally, data and analytics from ICU registries support informed decision-making for policymakers and healthcare administrators by providing data on ICU utilization, cost-effectiveness, and patient outcomes.^(4)^ Registries can also facilitate global collaboration by enabling international benchmarking and multicenter research, fostering a culture of continuous learning and improvement.^(5)^

Despite the growing importance of ICU registries for quality improvement (QI) and research, there is still a lack of consistency and reproducibility regarding the contents of the datasets. Different registries collect varying data sets using varied data structures, choose different predictive models, each with diverse definitions, and employ different methods for data collection and analysis. Understanding these variations is crucial to allow for the comparison of ICU practices and population outcomes between regions.^(6)^ By gaining insight into the specific datasets deployed by ICU registries around the globe, we can better identify uniformity and disparities, harmonize practices, and enhance the effectiveness of registries as tools for advancing critical care.^(7)^ Moreover, recent geo-economic analyses have underlined the differences in outcomes across ICUs worldwide, in different patient groups - underscoring the potential role of registries in streamlining these variations.^(8-10)^ The coronavirus disease 2019 (COVID-19) pandemic showed the importance of standardizing datasets and models to ensure comparable and replicable comparisons among different countries and health systems. In addition, harmonized and, where possible, matched datasets for case mix and outcomes measures would benefit the growing investment in international research collaborations, specifically global platform trials, disease surveillance, and quality of care research.

The Linking of Global Intensive Care (LOGIC) network is an initiative that globally connects ICU registries to enhance patient outcomes in critical care by promoting regional, national, and international benchmarking of ICU outcomes and harmonized research methods.^(5)^ In this study, Global mapping of ICU Registry Datasets (GRID), we aimed to map current core datasets collected by ICU registries, for benchmarking, resource utilization, service provision, QI, and clinical research.

## METHODS

### Study design

The study was designed as a cross-sectional survey disseminated to registry leads between October 2023 and June 2024. Ethical approval for the survey was not sought. Participating registries internationally have ethical approvals in place for the datasets they collect. The study is reported using the recommendations given in the Checklist for Reporting Results of Internet E-surveys (CHERRIES, detailed in  Table 1S - Supplementary Material).^(11)^

### Survey

A closed survey was developed by the LOGIC network. Three authors developed the first draft, with revisions by other four authors before review and approval from all co-authors. The survey was delivered using the online tool (Google Forms), and no incentives were provided. It was structured into four topics: (1) registry characteristics and coverage; (2) core dataset; (3) additional population or process-specific datasets; and (4) registry-enabled or registry-embedded research. The core dataset was defined as a set of variables collected in all ICU encounters, which enable the description of patient demographics and case mix and the benchmarking of risk-adjusted clinical outcomes. The survey responses were constructed from binary, multiple-choice questions with limited free text. Respondents had the opportunity to add narrative comments, detailing information on the QI and research initiatives. Respondents were also invited to share or signpost public links to guidance documents and data collection tools. A full version of the survey is available in  table 2S (Supplementary Material). Duplicate entries from the same national registry were harmonized, and investigators were directly in contact with them in case of discrepancies.

### Procedure

The closed survey was sent to registry leads in October 2023, purposely sampled through the email list comprising registries that are a part of LOGIC, and also an additional list of known ICU registries identified in a previous review,^(1)^ with the invitation to complete the survey only once per national registry. In case of incomplete responses, the responsible registry lead was contacted, and missing information was sought. Cleaned responses were shared with registry leads for verification.

### Statistical analysis

Survey results were described using percentages for categorical variables and median and interquartile range (IQR) for continuous variables, assuming non-normal distribution. Because of the study's descriptive nature, no association tests were conducted. Graphs were constructed using Graphpad Prism software (version 10.4, GraphPad Software, LLC).

## RESULTS

### Registries, characteristics, and coverage

**Figure 1:**
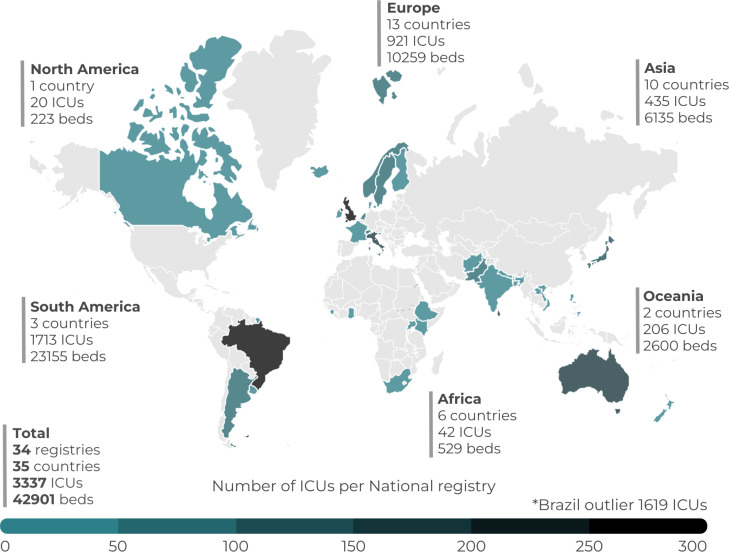
Heatmap showing the absolute number of intensive care units for the registries mapped in the current survey.

Of 42 invited registry leads, after harmonization of four duplicates, 34 registries were included in the final analysis (response rate 80%). Participating registries are operational in 35 countries, and are actively collecting data from 3,337 ICUs, accounting for more than 42,000 beds (Figure 1). Approximately half of the registries’ ICUs were in South America. There was a predominance of public ICUs (median 93% IQR 40 - 100%) with a median coverage of 50% (IQR 19 - 100%) of the country's ICUs (Table 1).

ICU - intensive care unit.

**Table 1:** Intensive care unit registries characteristics

Country	Registry name	ICUs (n)	Beds (n)	Proportion of public ICUs (%)	Coverage (%)*
Afghanistan	RICA†	40	425	69	NA
Argentina	SATI-Q	70	811	41	7
Australia & New Zealand	ANZICS CORE	206	2,600	93	92
Bangladesh	BACIR†	5	53	40	NA
Brazil	Epimed Monitor ICU Database	1,619	22,069	39	45
Canada	eCritical Alberta‡	20	223	100	100‡
Denmark	Danish Intensive Care Database	41	380	100	100
England	ICNARC	289	4,406	93	95
Ethiopia	Ethiopia ICU Registry†	5	60	100	NA
Finland	Finnish Intensive Care Consortium	27	270	100	100
France	CUB-Réa	30	500	100	10
Ghana	Ghana Critical Care Registry†	7	34	100	50
Iceland	Icelandic Intensive Care Registry	2	14	80	80
India	Indian Registry of Intensive Care (IRIS)^	19	1,018	11	NA
Ireland	Irish National ICU Audit	29	313	100	89
Italy	PROSAFE - GiViTi§	157	1,320	95	18
Japan	JIPAD	121	1,411	74	20
Kenya	Kenya Critical Care Registry†	8	94	38	11
Laos	Mahosot ICU†	1	12	22	5
Nepal	NICRF†	20	403	38	NA
Netherland	NICE	84	1,050	100	100
Norway	NIPaR	67	250	100	88
Pakistan	PRICE†	67	1,621	60	30
Philippines	PROTECT platform†	1	18	0	0.3
Scotland	SICSAG	22	340	100	100
South Africa	South Africa ICU Registry†	6	33	100	NA
Sierra Leone	Part of the CCAA network†	2	8	100	NA
Slovenia	PROSAFE International#	6	41	100	NA
Sri Lanka	NICS	155	982	100	NA
Sweden	SIR	82	465	100	100
Switzerland	SGI-SSMI	85	910	90	100
Uganda	Intensive Care Registry of Uganda†	14	300	25	50
Uruguay	UCIs Uruguayas	24	275	17	47
Vietnam	Vietnam ICU Registry†	6	192	83	NA

ICU - intensive care unit; RICA - Registry of Intensive Care of Afghanistan; NA - not applicable; SATI-Q - *Programa de Calidad de la Sociedad Argentina de Terapia Intensiva*; ANZICS CORE - Australia and New Zealand Intensive Care Society Centre for Outcome and Resource Evaluation; BACIR - Bangladesh Acute Care and ICU Registry; ICNARC - Intensive Care National Audit and Research Centre; CUB-Réa - *Collège des Utilisateurs des Bases des données en Réanimation*; PROSAFE - Promoting Patient Safety and Quality Improvement in Critical Care software; GiViTi - *Gruppo Italiano per la Valutazione degli Interventi in Terapia Intensiva*; JIPAD - Japanese Intensive care PAtient Database; NICRF - Nepal Intensive Care Research Foundation; NICE - Netherlands Intensive Care Evaluation; NIPaR - Norwegian Intensive Care and Pandemic Register; PRICE - Pakistan Registry of Intensive Care; SICSAG - Scottish Intensive Care Society Audit Group; CCAA - Collaboration for Research, Implementation and Training in Critical care in Asia and Africa; NICS - National Intensive Care Surveillance; SIR - Swedish Intensive Care Registry; SGI-SSMI - Swiss Society of Intensive Care Medicine; UCI - *Unidades de Cuidados Intensivos*.

* Estimated proportion of intensive care units in the country participating in the intensive care unit registry program;

† registries use the PROTECT platform currently curated by the CCAA network;

‡ Provincial rather than National clinical information system, hence coverage is mirroring the Alberta region;

§ PROSAFE International is curated by PROSAFE-GiViTi (Italy).

Intensive care unit registry data were submitted by dedicated data collectors or medical staff such as physicians, residents, or nurses; less than half of the data were automatically uploaded by electronic medical records integration. In most cases, patients’ consent was implied at the time of hospital admission as regulated by ethical review or national regulations (Table 2).

**Table 2:** Severity of illness, organ dysfunction scores, additional modules implemented in national intensive care unit registries, and other registries structural characteristics

Scoring systems			Registries (n = 34)
Severity of illness			
	SAPS II		9 (26.5)
	SAPS 3		4 (11.8)
	APACHE II		23 (67.6)
	APACHE III		4 (11.8)
	APACHE IV		3 (8.8)
	eTropICS		14 (29.4)
	Own risk prediction model		7 (20.6)
	Other (e.g., PIM)		3 (8.8)
Organ dysfunction*			
	SOFA		30 (88.2)
		Categorical numerical values	22 (64.7)
		Categorical numerical ranges	6 (17.6)
		Only total score	2 (5.9)
	None		4 (11.8)
Type of additional modules/datasets			
	Staffing modules		11 (32.3)
	Bed availability		9 (26.5)
	Trauma		3 (8.8)
	ICU-acquired infections		21 (58.8)
	Colonization module		4 (11.8)
	Cardiothoracic		6 (17.6)
	Other		8 (23.5)
	None		5 (14.7)
Data collection process			
	Dedicated data collector		27 (79.4)
	Automatic integration with EMR		17 (50.0)
	Medical staff's free time		
		Physicians or residents	18 (52.9)
		Nurses	12 (35.3)
	Other		6 (17.6)
Method of informed consent			
	Individual patient consent for any registry input		3 (8.8)
	Individual patient consent only for embedded research		3 (8.8)
	Other		4 (11.8)
	Waiver†		24 (72.7)

SAPS - Simplified Acute Physiology Score; APACHE - Acute Physiology and Chronic Health Evaluation; eTropICS - Electronic Tropical Intensive Care Score; PIM - Pediatric Index of Mortality; SOFA - Sequential Organ Failure Assessment; ICU - intensive care unit; EMR - electronic medical record.

* Non-exclusive categories, i.e., national registries, may have more than one item;

† individual consent was waived by ethical review or national regulations. Data presented as n (%).

### Nomenclature, diagnostic coding systems, and risk prediction models

**Figure 2:**
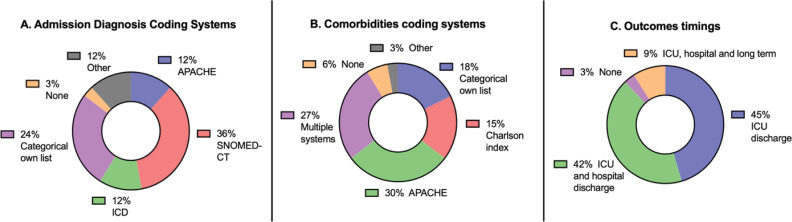
Main diagnostic coding systems (panel A), comorbidities coding systems (panel B), and timing of outcomes (panel C).

**Figure 3:**
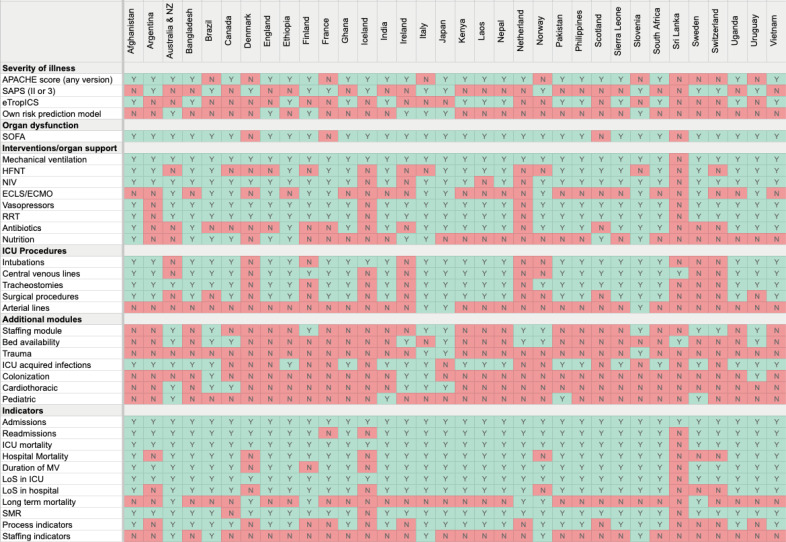
Heatmap on reported interventions, procedures, additional modules, and quality of care indicators.

There was a significant variability in nomenclatures, diagnostic coding, and prognostic models used by the registries in their core data sets. Systematized Nomenclature of Medicine Clinical Terms (SNOMED-CT) and customized categorical classifications were the main nomenclature and diagnostic coding systems, followed by the use of the diagnostic lists of the Acute Physiology and Chronic Health Evaluation II (APACHE II) coding and International Classification of Diseases (ICD, Figure 2A). For comorbidities, the APACHE II score list of comorbidities was currently adopted in > 50% of mapped ICU registries, followed by customized lists and the conditions that constitute the Charlson comorbidity index (Figure 2B and 3). Regarding the severity of illness scores and risk prediction models, 33 out of 34 (97%) registries reported using at least one, with the APACHE II score being the most frequently used (Table 3). One out of five registries used an in-house mortality prediction model developed explicitly by their national teams. The Sequential Organ Failure Assessment (SOFA) score was collected by 30 (88%) of registries, with the remaining registries not currently measuring any organ dysfunction score.

APACHE - Acute Physiology and Chronic Health Evaluation; SNOMED-CT - Systematized Nomenclature of Medicine Clinical Terms; ICD - International Classification of Diseases; ICU - intensive care unit.

APACHE - Acute Physiology and Chronic Health Evaluation; SAPS - Simplified Acute Physiology Score; eTropICS - Electronic Tropical Intensive Care Score; SOFA - Sequential Organ Failure Assessment; HFNT - high flow nasal therapy; NIV - noninvasive ventilation; ECLS/ECMO - extracorporeal life support/extracorporeal membrane oxygenation; RRT - renal replacement therapy; ICU - intensive care unit; MV - mechanical ventilation; LoS - length of stay; SMR - standardized mortality rate;

For procedures, some national registries record both the intervention (e.g., endotracheal tube, central venous line, tracheal cannula) and duration, while others only record the event.

A live version of this heatmap will be available on the Linking of Global Intensive Care (LOGIC) website:- www.icubenchmarking.com

**Table 3:** Types of organ support, procedures and methods for sedation assessment across registries

				Registries (n = 34)
Organ support				
	Respiratory support			
		Invasive mechanical ventilation		33 (97.1)
		NIV		29 (85.3)
		HFNC		21 (61.8)
		ECMO		12 (35.3)
	Hemodynamic support			30 (85.7)
		Blood purification techniques		
			Renal replacement therapy	30 (85.7)
			Plasmapheresis	2 (5.9)
	Procedures			
		Intubations		25 (73.5)
		Tracheostomy		26 (76.5)
		Central venous lines		26 (76.5)
		Arterial lines		3 (8.8)
		Urinary catheter		3 (8.8)
	Other interventions			
		Nutrition		10 (29.4)
		Antibiotics		19 (55.9)
		Surgical procedures		21 (61.8)
Sedation assessment				
	No sedation assessment			13 (38.2)
	Measured and Target RASS			14 (41.2)
	Measured RASS			3 (8.8)
	Other methods			4 (11.8)

NIV - noninvasive ventilation; HFNC - high flow nasal cannula; ECMO - extracorporeal membrane oxygenation; RASS - Richmond agitation sedation scale. Data presented as n (%).

### Other core dataset characteristics

Intensive care unit interventions and procedures reported in ICU registries are detailed in figure 3. The use of organ support measures was widely tracked in core datasets, led by invasive mechanical ventilation (97%, with duration of ventilation reported in 88% of registries), haemodynamic support (86%), and renal replacement therapy (86%, Figure 3 and Table 3). Three out of four registries collect procedures and interventions performed on patients, including intubations, invasive lines, tracheostomies, and organ support or treatment given to patients; however, the captured procedures vary substantially among registries (Figure 3 and Table 4). When sedation assessment was performed, the Richmond Agitation-Sedation Scale (RASS) was used most commonly. In terms of ICU indicators all registries coded ICU admissions and 91% recorded readmissions. The standardized mortality rate was computed by most registries (91%), and process indicators were used in 65% of registries (Figure 3 and  Table 3S [Supplementary Material]).

**Table 4:** Types of registry-enabled quality improvement initiatives

Feature		Registries (n = 34)
Quality improvement initiative*		22 (64.7)
	Quality improvement toolkits	4 (11.8)
	Outlier programs	6 (17.6)
	Flagging of potential QI targets	6 (17.6)
	Data communication to the government or ministry	11 (32.4)
	Other	2 (5.9)
	None	12 (35.3)

QI - quality improvement.

* Non-exclusive categories. Data presented as n (%).

### Additional datasets and initiatives

In addition to the core dataset, ICU registries provide several additional modules that differ widely between countries. The most frequently adopted additional modules were bed availability, surveillance of ICU-acquired infections and ICU staffing reporting (Figure 3 and Table 2). Over half of ICU registries reported structured QI initiatives guided by the registry team. Recurrent reported initiatives were QI toolkits, QI outlier programs, and data communication to government or public institutions (Table 4).

### Use of intensive care unit registries for registry-enabled or registry-embedded research

The percentages of registries that use or let data be used for observational or interventional research studies are 50.0% and 17.6%, respectively.

## DISCUSSION

### Main findings

This study provides new information on the spread and scope of ICU registry data sets, the characteristics, nomenclature, and prediction models used, and how such data is being applied for service evaluation, research, and QI. Interestingly, we were able to identify that fewer than 20% of the world's countries appear to have an active ICU registry. However, it is possible that some may not have been captured in our assessment. The GRID survey shows that ICU registries can be used to support QI initiatives, advancing patient care and research - while highlighting substantial variability in data collection, coding systems and operational scope. This variability presents both challenges and opportunities for the global critical care community. Despite challenges, ICU registries remain an invaluable resource for understanding and improving intensive care practices worldwide.^(12,13)^

We record an increasing structuring of ICU registries in low and middle-income countries in Asia and Africa.^(7,14,15)^ While some registries focus on providing case mix and outcomes for audit purposes, many ICU registries deliver additional information related to resource use, process of care, and QI initiatives, thereby collecting and processing more data.

We report a significant heterogeneity in the data collected by ICU registries, especially in terms of nomenclature, diagnostic coding systems, and comorbidities handling. Registries employ a mix of SNOMED-CT, customized classifications, and ICD-based systems. Such diversity reflects the differing priorities and resources available to registries but also underscores the necessity of harmonization to facilitate global benchmarking and collaboration. Differences in nomenclature can be overcome by strategies such as federated analysis, mapping common data models, and using established reference metadata standards. Examples of such standards are those established by the Clinical Data Interchange Standards Consortium^(16)^ - and the standard data model proposed by the Observational Medical Outcomes Partnership (OMOP).^(17)^ On the other hand, different definitions for outcome measures are more complex to unpick and may lead to misleading findings in benchmarking and clinical trials. Thus, promoting selected core outcome sets with standardized nomenclature, wider use of standard data models, and rigorous public availability of dictionaries and codebooks from the national registries, is important.

While some registries comprehensively capture interventions, severity scores, and procedural data, others are limited to case-mix and outcomes variables, omitting procedures or organ dysfunction metrics. For instance, although most registries use established severity scoring systems like APACHE II or SOFA, a modest portion collects no organ dysfunction score at baseline and even less do it longitudinally. This inconsistency complicates international comparisons and highlights the need for standardized data collection practices. The COVID-19 pandemic further highlighted the critical importance of standardized, high-quality data in supporting global health responses and tracking patient outcomes.^(18)^ Future registry development must incorporate these lessons, ensuring that ICU data systems are resilient, adaptable, and capable of addressing the challenges of future health crises.^(19)^

### Quality improvement and research

The survey also reveals the growing emphasis on QI initiatives within registries. Over half reported structured QI programs, including toolkits, outlier detection programs, and data communication to governmental bodies. These initiatives demonstrate the evolving maturity of ICU registries, which are increasingly recognized as tools for data collection that can inform systemic healthcare improvements.^(20–22)^ While the use of registries for QI initiatives is increasingly documented, whether these tools lead to actual changes in patient care will need to be further studied and documented.^(19)^

The limited integration of registries into research frameworks remains a missed opportunity. Interestingly, while more than 40% of registries support observational studies, only one out of five are involved in interventional research. Expanding the role of registries in research could unlock their full potential as platforms for generating real-world evidence and guiding clinical practice.

The lack of uniformity in core datasets and coding systems creates barriers to effective international benchmarking. Moreover, the absence of a comprehensive "registry of registries" complicates efforts to ensure inclusivity in mapping studies like GRID. Legal and logistical barriers, including informed consent and data interoperability issues, further constrain some registries’ functionality, particularly in resource-limited settings. Nevertheless, the findings of the GRID survey provide a roadmap for addressing these challenges. Global initiatives like the LOGIC network are well-positioned to drive efforts toward standardization.^(5)^ LOGIC and similar organizations can facilitate meaningful comparisons between registries and enhance their utility in global collaborations by promoting standard data models, harmonized coding systems, and interoperable frameworks. Such efforts could also support emerging registries in low- and middle-income countries, fostering their development through technical assistance and capacity building.

### Strengths and limitations

The strength of this survey lies in its novelty in attempting to map core features of existing national registries worldwide. Spreading the survey through an existing consortium such as LOGIC allowed to increase the reach of the exploration. This survey had several limitations. To our knowledge, we contacted all existing National or regional ICU registries worldwide. Since there is no such thing as a registry of registries, we were unable to verify the completeness of the registries included. In this mapping study using a questionnaire sent out by mail to ICU registry leads, we were unable to verify the answers using the actual datasets of the registries. Differences in interpreting the posed questions could have led to interpretation errors in our analysis. The study's results were shared with all leads to check the answers they gave, and minor corrections were made to the data. Although we reported on consent requirements, we did not explore potential barriers to registry functionality, such as legal, cultural, and policy barriers preventing the data collection, use, sharing, or analysis. In addition, we did not explore whether organ support measures were tracked for resource utilization or care processes, although this has implications for the cadence of data collection.

## CONCLUSION

While the GRID survey reveals considerable variability in intensive care unit registries, it also highlights their increasing global spread and potential as quality improvement, research, and policy development tools. Addressing the challenges of standardization, interoperability, and resource constraints will be crucial for harnessing registries’ full potential. By fostering global collaboration and advancing the integration of registries into research and quality improvement initiatives, the critical care community can drive significant improvements in patient care and outcomes worldwide.

## GRID INVESTIGATORS OF THE LINKING OF GLOBAL INTENSIVE CARE (LOGIC) (18)

Christina Agvald-Öhman: Anaesthesiology and Intensive Care, Karolinska University Hospital Huddinge - Stockholm, Sweden; John Amuasi: Department of Global Health, Kwame Nkrumah University of Science and Technology - Kumasi, Ghana; Stepani Bendel: Finnish Intensive Care Consortium - Finland; Christian F. Christiansen: Danish Intensive care Database (DID) - Denmark; Arjen Dondorp: Mahidol Oxford Tropical Research Unit - Bangkok, Thailand; Guilherme Cortes Fernandes: Epimed - Brazil; Aniruddha Ghose: Department of Medicine, Chittagong Medical College Hospital - Chattogram, Bangladesh; Johnny Hillgren: Swedish Intensive Care Registry - Sweden; David Litton: Australian and New Zealand Intensive Care Society (ANZICS) Centre for Outcome and Resource Evaluation Clinical Quality Registry - Australia, New Zealand; Sruthi Mano: Indian Registry of IntenSive care (IRIS) - India; Chamira Kodippily and Dilanthi Priyadarshani: Nat-Intensive Care Surveillance, Mahidol Oxford Tropical Medicine Research Unit - Colombo, Sri Lanka; Kathy Rowan: Intensive Care National Audit & Research Centre - United Kingdom; Carolyne Njoki and Eunice Tole: Aga Khan University Hospital - Nairobi, Kenya; Adam H. Smit: Faculty of Health Sciences, Busitema University - Mbale, Uganda and William Harvey Research Institute, Queen Mary University of London - United Kingdom; Marcio Soares: *Instituto D’Or de Pesquisa e Ensino* - Rio de Janeiro, Brazil.

## Data Availability

Data will be available upon request.
