# Supplementary material for: Characteristics of intensive care unit registries - findings from the Global Registry ICU Datasets (GRID) survey
Source: Crit Care Sci. 2026 Jan 9;38:e20260168. doi: 10.62675/2965-2774.20260168 (PMC12977222; doi:10.62675/2965-2774.20260168)
Supplement: Supplementary Material [file 2965-2774-ccsci-38-e20260168-suppl1.pdf]

Critical Care Science
https://doi.org/10.62675/2965-2774.20260168

Characteristics of intensive care unit registries - findings from the Global Registry ICU Datasets (GRID) survey

Luigi Pisani[1,2], Paola Di Lecce[1], Cornelius Sendagire[3], Vrindha Pari[4], Carlo Olivieri[5], Rabiul Alam Md Erfam Uddin[6], Diptesh Aryal[7], Priyantha Athapattu[8], Sean Bagshaw[9], Gaston Burghi[10], Eirik Alnes Buanes[11,12], Steffen Christensen[13], Rory Dwyer[14], Ariel Leonardo Fernández[15], Stefano Finazzi[16], Bertrand Guidet[17], David Harrison[18], Eva Hanciles[19], Madiha Hashmi[20], Satoru Hashimoto[21], Nao Ichihara[21], Nazir I. Lone[22,23], Maria del Pilar Arias López[15], Yen L. Minh[24], Andreas Perren[25], Koukeo Phommasone[26], David Pilcher[27], Matti Reinikainen[28], Wangari Waweru-Siika[29], Moses Siaw-Frimpong[30], Martin I. Sigurdsson[31], Maryam Shamal[32], Menbeu Sultan[33], Jose Emmanuel M. Palo[34], David Thomson[35], Bharath Kumar Tirupakuzhi Vijayaraghavan[36], Abigail Beane[2], Rashan Haniffa[22], Dave A. Dongelmans[37], Miklos Lipcsey[38], Jorge Ibrain Figueira Salluh[39] for the GRID investigators of the Linking of Global Intensive Care (LOGIC)

Table 1S - Checklist for Reporting Results of Internet E-Surveys (CHERRIES)

Checklist item	Explanation	Section
Describe survey design	Describe target population, sample frame. Is the sample a convenience sample? (In "open" surveys this is most likely)	Methods
IRB approval	Mention whether the study has been approved by an Institutional Review Board	Methods
Informed consent	Describe the informed consent process. Where were the participants told the length of time of the survey, which data were stored and where and for how long, who the investigator was, and the purpose of the study?	NA
Data protection	If any personal information was collected or stored, describe what mechanisms were used to protect unauthorized access.	NA
Development and testing	State how the survey was developed, including whether the usability and technical functionality of the electronic questionnaire had been tested before fielding the questionnaire.	Methods
Open survey versus closed survey	An "open survey" is a survey open for each visitor of a site, while a closed survey is only open to a sample which the investigator knows (password-protected survey).	Methods
Contact mode	Indicate whether or not the initial contact with the potential participants was made on the Internet. (Investigators may also send out questionnaires by mail and allow for Web-based data entry)	Methods
Advertising the survey	How/where was the survey announced or advertised? Some examples are offline media (newspapers), or online (mailing lists – If yes, which ones?) or banner ads (Where were these banner ads posted and what did they look like?). It is important to know the wording of the announcement as it will heavily influence who chooses to participate. Ideally the survey announcement should be published as an appendix.	NA
Web/E-mail	State the type of e-survey (eg, one posted on a Web site, or one sent out through e-mail). If it is an e-mail survey, were the responses entered manually into a database, or was there an automatic method for capturing responses?	Methods
Context	Describe the Web site (for mailing list/newsgroup) in which the survey was posted. What is the Web site about, who is visiting it, what are visitors normally looking for? Discuss to what degree the content of the Web site could pre-select the sample or influence the results. For example, a survey about vaccination on a anti-immunization Web site will have different results from a Web survey conducted on a government Web site	NA
Mandatory/ voluntary	Was it a mandatory survey to be filled in by every visitor who wanted to enter the Web site, or was it a voluntary survey?	Methods
Incentives	Were any incentives offered (eg, monetary, prizes, or non-monetary incentives such as an offer to provide the survey results)?	NA
Time/date	In what timeframe were the data collected?	Methods
Randomization of items or questionnaires	To prevent biases items can be randomized or alternated.	NA
Adaptive questioning	Use adaptive questioning (certain items, or only conditionally displayed based on responses to other items) to reduce number and complexity of the questions.	NA
Number of Items	What was the number of questionnaire items per page? The number of items is an important factor for the completion rate.	Methods

Continue...

This is an open access article under the CC BY license (https://creativecommons.org/licenses/by/4.0/).

...continuation

Checklist item	Explanation	Section
Number of screens (pages)	Over how many pages was the questionnaire distributed? The number of items is an important factor for the completion rate.	NA
Completeness check	It is technically possible to do consistency or completeness checks before the questionnaire is submitted. Was this done, and if "yes", how (usually JAVAScript)? An alternative is to check for completeness after the questionnaire has been submitted (and highlight mandatory items). If this has been done, it should be reported. All items should provide a non-response option such as "not applicable" or "rather not say", and selection of one response option should be enforced.	NA
Review step	State whether respondents were able to review and change their answers (eg, through a Back button or a Review step which displays a summary of the responses and asks the respondents if they are correct).	Methods
Unique site visitor	If you provide view rates or participation rates, you need to define how you determined a unique visitor. There are different techniques available, based on IP addresses or cookies or both.	NA
View rate (ratio of unique survey visitors/ unique site visitors)	Requires counting unique visitors to the first page of the survey, divided by the number of unique site visitors (not page views!). It is not unusual to have view rates of less than 0.1 % if the survey is voluntary.	NA
Participation rate (ratio of unique visitors who agreed to participate/ unique first survey page visitors)	Count the unique number of people who filled in the first survey page (or agreed to participate, for example by checking a checkbox), divided by visitors who visit the first page of the survey (or the informed consents page, if present). This can also be called "recruitment" rate.	Results
Completion rate (Ratio of users who finished the survey/users who agreed to participate)	The number of people submitting the last questionnaire page, divided by the number of people who agreed to participate (or submitted the first survey page). This is only relevant if there is a separate "informed consent" page or if the survey goes over several pages. This is a measure for attrition. Note that "completion" can involve leaving questionnaire items blank. This is not a measure for how completely questionnaires were filled in. (If you need a measure for this, use the word "completeness rate".)	Results
Cookies used	Indicate whether cookies were used to assign a unique user identifier to each client computer. If so, mention the page on which the cookie was set and read, and how long the cookie was valid. Were duplicate entries avoided by preventing users access to the survey twice; or were duplicate database entries having the same user ID eliminated before analysis? In the latter case, which entries were kept for analysis (eg, the first entry or the most recent)?	Not done
IP check	Indicate whether the IP address of the client computer was used to identify potential duplicate entries from the same user. If so, mention the period of time for which no two entries from the same IP address were allowed (eg, 24 hours). Were duplicate entries avoided by preventing users with the same IP address access to the survey twice; or were duplicate database entries having the same IP address within a given period of time eliminated before analysis? If the latter, which entries were kept for analysis (eg, the first entry or the most recent)?	Not done
Log file analysis	Indicate whether other techniques to analyze the log file for identification of multiple entries were used. If so, please describe.	Methods
Registration	In "closed" (non-open) surveys, users need to login first and it is easier to prevent duplicate entries from the same user. Describe how this was done. For example, was the survey never displayed a second time once the user had filled it in, or was the username stored together with the survey results and later eliminated? If the latter, which entries were kept for analysis (eg, the first entry or the most recent)?	Not done
Handling of incomplete questionnaires	Were only completed questionnaires analyzed? Were questionnaires which terminated early (where, for example, users did not go through all questionnaire pages) also analyzed?	Methods
Questionnaires submitted with an atypical timestamp	Some investigators may measure the time people needed to fill in a questionnaire and exclude questionnaires that were submitted too soon. Specify the timeframe that was used as a cut-off point, and describe how this point was determined.	NA
Statistical correction	Indicate whether any methods such as weighting of items or propensity scores have been used to adjust for the non-representative sample; if so, please describe the methods.	NA

Source: modified from: Eysenbach G. Improving the quality of Web surveys: the Checklist for Reporting Results of Internet E-Surveys (CHERRIES). J Med Internet Res. 2004 Sep 29;6(3):e34. Erratum in J Med Internet Res. 2012;14(1):e8.[11]

Table 2S - Global mapping of Registries ICU Datasets (GRID)

Global mapping of Registries ICU Datasets (GRID)

* Indicates required question

1. E-mail*

____

2. Registry name*

____

3. Registry website (if available)

____

4. Number of participating ICUs*

____

5. Number of ICU beds (total)

____

6. Proportion of PUBLIC ICUs (%)

____

7. Estimated coverage (if known)

   I.e. Percentage *of ICUs in the registry over total ICUs in the country or region*

____

8. What patient-level consent method do you use?*

   If you choose other, please detail

   Mark only one oval.

◯ None, individual consent was waived by ethical review or national regulations

◯ Individual patient consent for any registry input

◯ Individual patient consent only for embedded research

◯ Other: ____

9. How can users access their site data? *

   If you choose other, please detail

   Check all that apply

☐ No direct access

☐ Online dashboard

☐ PDF report

☐ Analyzer on online platform

☐ Specific reports by request

☐ Other: ____

10. Name of the registry platform (if different from registry name)

____

11. Does the registry platform perform automatic quality of data checks

Mark only one oval

◯ No

◯ Yes

12. Does the registry team perform any form of source data verification? e.g. on a small subset of patients

Mark only one oval

◯ Yes

◯ No

Skip to question 13

CORE DATASET

This section seeks to understand how the core dataset of your registry is built and what variables it contains. By core data set we mean "data that is mandatory for all ICUs in the registry"

13. Main diagnostic coding system *

If you choose other, please detail

Mark only one oval

◯ Categorical own list

◯ ICD classification

◯ Snomed CT

◯ APACHE library

◯ Other: ____________________

14. Comorbidities coding system*

Check all that apply, and give details if 'other'

Check all that apply

◯ None, comorbidities are not collected

◯ Own list of comorbidities

◯ Charlson index

◯ APACHE comorbidities

◯ Other: ____________________

15. Severity of illness scoring systems*

Check all that apply, and give details if 'other'

Check all that apply

☐ None

☐ SAPS 2

☐ SAPS 3

☐ APACHE II

☐ APACHE III

☐ APACHE IV

☐ GIVITI model

☐ ICNARC

☐ ANZROD

☐ JIPAD
☐ EPM (Epimed)
☐ Other: ____________________

16. For the assessment of scoring systems you collect (check all that apply)*

Check all that apply

☐ Scores as global values calculated offline
☐ All physiologic, lab and clinical variables that make the score (numerical)
☐ All physiologic, lab and clinical variables that make the score (categorical, only RANGE)

17. Organ dysfunction scoring systems*

Check all that apply, and give details if 'other'

Check all that apply

☐ None
☐ SOFA as total number
☐ SOFA as individual categories (numerical values)
☐ SOFA as individual categories (only ranges)
☐ LODS
☐ Other: ____________________

18. Organ support and treatments covered in the CORE*

Check all that apply, and give details if 'other'

Check all that apply

☐ Invasive mechanical ventilation
☐ NIV
☐ High flow nasal therapy (HFNT)
☐ ECLS & ECMO
☐ Renal replacement therapy
☐ Vasopressors
☐ Antibiotics
☐ Nutrition (enteral/parenteral)
☐ Other: ____________________

19. Procedures covered*

Check all that apply, and give details if 'other'

Check all that apply

☐ None
☐ Intubations
☐ Central venous lines
☐ Tracheostomy
☐ Surgical procedures
☐ Other: ____________________

20. Sedation assessmen*

Check all that apply, and give details if 'other'

Check all that apply

☐ None
☐ Measured RASS (or other sedation score)
☐ Target RASS (or other sedation score)
☐ Sedative drugs used
☐ Other: ____

21. Is a data dictionary or data entry guidelines available to users?*

Mark only one oval

◯ Yes (please send file!)
◯ No

22. Does the CORE dataset include daily data collection i.e. repeated variables during ICU* stay

Mark only one oval

◯ Yes (please send file of daily variables)
◯ No

23. Any additional detail on repeated variables during ICU stay (e.g. proportion of ICUs using them, etc.)

____
____

24. Patient outcomes are collected at*

Mark only one oval

◯ ICU discharge
◯ Hospital discharge
◯ Other: ____

ADDITIONAL MODULES AND INDICATORS

25. Which additional modules does your registry have?*

Check all that apply, and give details if ‘other’

Check all that apply

☐ None
☐ Staffing module
☐ ICU bed availability
☐ ICU acquired infections
☐ Colonization module
☐ Cardiothoracic
☐ Trauma module
☐ Other: ____

26. If other, please specify

____
____

27. Is there any structured QUALITY IMPROVEMENT initiative guided by the registry team?*

Check all that apply, and give details if ‘other’

Check all that apply

- ☐ None
- ☐ Quality improvement toolkits
- ☐ Outlier program
- ☐ Flagging of potential QI targets
- ☐ Data communication to government or Ministry
- ☐ Other: ____________________

28. Please give details on the QI initiative(s) flagged above

____

____

29. What INDICATORS are regularly provided or accessible to the sites?*

Check all that apply, and give details if 'other'

Check all that apply

- ☐ ICU mortality
- ☐ Hospital mortality
- ☐ Long term mortality (please specify timepoints below)
- ☐ Functional outcomes at discharge (please detail below)
- ☐ Length of stay, ICU
- ☐ Length of stay, hospital
- ☐ SMR standardize mortality rate
- ☐ SRU standardized resources use
- ☐ Number of admissions
- ☐ Number of REadmissions
- ☐ Duration of MV
- ☐ Process indicators (please detaile below)
- ☐ Staffing indicators (please detail below)
- ☐ Other: ____________________

30. Please give additional details on indicators or provide list as attachment

____

____

31. If Long term mortality was chosen, please specify timepoints

____

____

32. Usually, how is the registry data collected in the different ICUs?*

Check all that apply, and give details if 'other'

Check all that apply

- ☐ Dedicated data collector
- ☐ Automatic integration with EMR and other hospital systems
- ☐ Physicians and resident free time
- ☐ Nurses in free time
- ☐ Other: ____________________

33. If integration with electronic medical records (EMR) exist, please give details

____

REGISTRY BASED RESEARCH

34. Are there currently multicenter OBSERVATIONAL studies being enabled or embedded in the registry?*

Mark only one oval

◯ No

◯ Yes, please detail (name of project, if registered, planned substudies)

35. If you clicked yes to the question above, please detail OBSERVATIONAL studies (name of project, if registered, planned substudies)

____

____

36. Are there currently multicenter INTERVENTIONAL studies being enabled or embedded in the registry?*

Mark only one oval

◯ No

◯ Yes, please detail (name of project, if registered, planned substudies)

37. If you clicked yes to the question above, please detail INTERVENTIONAL studies (name of project, if registered, planned substudies)

____

____

Skip to question 38

DOCUMENTS SHARED FOR GRID STUDY

38. Please list all documentation you are sharing for the global mapping of ICU registry datasets study

Mark only one oval per row

	PDF or other file type	Link to website
List of CORE variables	◯	◯
List of daily (repeated) variables	◯	◯
List of additional modules variables	◯	◯
Data entry guidelines	◯	◯
Additional modules description	◯	◯
National registry description	◯	◯
Methods paper	◯	◯
Registry quality evaluation	◯	◯
Example of registry report	◯	◯
Example of toolkit or ICU QI suport	◯	◯

This content is neither created nor endorsed by Google.
Google Forms

Table 3S - Indicators collected by the intensive care unit registries

Indicators	Number of registries (n = 34)
Mortality	
ICU	33 (97.1)
Hospital	27 (79.4)
Long term	6 (17.6)
SMR	31 (91.2)
Length of stay	
ICU	33 (97.1)
Hospital	27 (79.4)
Number of	
Admissions	34 (100)
Readmissions	31 (91.2)
Other indicators	
Duration of MV	30 (88.2)
Process indicators	22 (64.7)
Staffing indicators	5 (14.7)

ICU - intensive care unit; SMR - standardized mortality rate; MV - mechanical ventilation. Data presented as n (%).
